# Effects of 12-week Circuit Exercise Intervention on Blood Pressure, Vascular Function, and Inflammatory Cytokines in Obese Older Women with Sarcopenia

**DOI:** 10.31083/j.rcm2505185

**Published:** 2024-05-22

**Authors:** Won-Sang Jung, Hana Ahn, Sung-Woo Kim, Hun-Young Park

**Affiliations:** ^1^Department of Senior Exercise Prescription, Dongseo University, 47011 Busan, Republic of Korea; ^2^Physical Activity and Performance Institute (PAPI), Konkuk University, 05029 Seoul, Republic of Korea; ^3^Department of Sports Medicine and Science, Graduated School, Konkuk University, 05029 Seoul, Republic of Korea

**Keywords:** circuit exercise, blood pressure, vascular function, inflammatory cytokines, obese, sarcopenia, older women

## Abstract

**Background:**

This study investigates the effects of a 12-week circuit exercise program on blood pressure, vascular function, and inflammatory cytokines in older obese women with sarcopenia.

**Methods:**

Twenty-eight older obese women with sarcopenia (mean age: 78.2 ± 3.7 years) were randomly divided into an exercise group (EG, n = 14) and a control group (CG, n = 14). The EG participated in a 12-week circuit exercise training regimen, conducted three times weekly, with each session lasting between 45 to 75 minutes (progressively increased over time). The CG was advised to maintain their regular daily routines throughout the intervention period. All dependent variables, including blood pressure, vascular function, and inflammation cytokines, were evaluated pre- and post-intervention.

**Results:**

Positive changes were observed in the EG in body composition (body fat mass; *p* < 0.001, body fat percentage; *p* < 0.01, free-fat mass; *p* < 0.01), blood pressure (heart rate; *p* < 0.05, rate pressure product; *p* < 0.01), vascular function (brachial–ankle pulse wave velocity; *p* < 0.05, flow-mediated dilation; *p* < 0.001), and inflammation cytokines (interleukin-6; *p* < 0.05). In the CG, there was an increase in body fat mass (*p* < 0.05) and body fat percentage (*p* < 0.05), while no changes were observed in other variables.

**Conclusions:**

The 12-week circuit exercise program significantly reduced blood pressure, improved vascular function, and decreased inflammatory cytokines in obese older women with sarcopenia. However, individual variations in response highlight the need for personalized exercise regimens.

## 1. Introduction

During aging, most physiological functions change, notably a decrease in lean body mass and muscle strength, alongside an increase in body fat [1]. This condition, sarcopenia, is characterized by an age-related reduction in muscle mass, strength, and function [2]. Older individuals are particularly susceptible to obesity due to this concurrent increase in body fat, leading to a heightened risk of various negative clinical outcomes such as endocrine, metabolic, and cardiovascular diseases [3, 4]. These conditions contribute to increased healthcare costs and declining quality of life [5]. Furthermore, reports suggest that women may experience a greater decline in physical function and a higher risk of diseases compared to men [6].

Aging leads to a decline in physical functions, and limited physical activity often accompanies obesity, resulting in an increase in obese individuals with sarcopenia [7]. It has been reported that the risk of metabolic and cardiovascular diseases is closely related to aging. Obesity is a significant factor in various lifestyle-related diseases, including hypertension, diabetes, and hyperlipidemia, and these conditions often lead to cardiovascular disease [8]. Previous studies indicate that high cholesterol and triglycerides accumulate on the inner walls of blood vessels, reducing their elasticity and leading to the narrowing and blockage of these vessels, a condition known as atherosclerosis [3, 9].

Hypertension arises from degenerative changes in the media—the middle layer of blood vessels—leading to fibrosis and reduced vascular elasticity as part of aging. This results in systolic hypertension, causing the heart muscle to thicken, a phenomenon known as cardiac hypertrophy [10]. The increase in peripheral vascular resistance, the stiffening of central arteries, and the early return of reflected waves lead to an increase in systolic blood pressure and a decrease in diastolic blood pressure [11]. These changes are significantly correlated with cardiovascular diseases.

Additionally, aging brings about various changes in the blood vessels, including increased arterial diameter, hypertrophy of the arterial wall, increased arterial stiffness, and endothelial dysfunction [12, 13, 14, 15, 16]. Furthermore, inflammation is described as a key mechanism in the development of atherosclerosis, with C-reactive protein (CRP), interleukins, tumor necrosis factor-alpha (TNF-α), and transforming growth factor-beta (TGF-β1) identified as risk factors [17, 18, 19]. Such sarcopenic obesity is closely associated with a systemic inflammatory state, necessitating the monitoring of inflammatory cytokines, such as interleukin (IL)-6 and CRP [20]. Therefore, given the significant risks associated with sarcopenic obesity in older adults, whose physical functions are already diminished by aging, efforts to prevent and improve sarcopenic obesity are crucial to mitigate the various diseases and physiological issues caused by obesity.

This study aims to verify the effectiveness of long-term circuit training in improving blood pressure, vascular function, and inflammatory markers in older obese women with sarcopenia. Previous studies have primarily conducted separate resistance and aerobic exercises to prevent sarcopenia and obesity, including resistance band exercises, isokinetic exercises, whole-body electronic muscle stimulation (EMS), and weight training. However, resistance exercises have been mostly recommended as a method for increasing muscle mass in obese older individuals with sarcopenia [21]. Recent studies have reported that a combination of aerobic and resistance exercises effectively reduces body weight and improves physical function in older individuals with sarcopenia and obesity [22]. Specifically, circuit training, which includes flexibility, aerobic, and resistance exercises, is recommended due to its low risk of injury, independence from cost or location, and ability to maintain continuous interest in exercise. As an exercise model, alternating between aerobic and resistance exercises in a continuous sequence, circuit training has been proven to effectively develop cardiovascular and muscular functions simultaneously [23, 24]. Jung *et al*. [25] analyzed the effects of a 12-week circuit exercise training program on body composition, physical fitness, muscle function, and lung function in older women with sarcopenia, revealing that such training effectively prevents sarcopenia and enhances physical fitness. This suggests that similar benefits could be observed in obese older women with sarcopenia. Moreover, Jung *et al*. [24] reported that the 12-week circuit training program positively influenced cardiovascular risk factors, vascular inflammation markers, and insulin-like growth factor 1 (IGF-1) improvements in older women with both sarcopenia and obesity. While our study involves similar subjects and variables, it uniquely incorporates flow-mediated dilation (FMD) to provide a more comprehensive understanding of the impact of exercise on blood pressure and vascular function in obese older individuals with sarcopenia. Park *et al*. [26] conducted a study on obese older men, demonstrating that a combined aerobic and resistance exercise program effectively improved body composition and cardiometabolic risk factors, such as blood pressure, arterial stiffness, and physical function. Although the previous study focused on older men, we anticipate that implementing a regular combined exercise program for obese older women with sarcopenia will yield beneficial health-related outcomes, similar to the previous research findings. In light of these prior studies, our research aims to elucidate and significantly advance the understanding of the physiological and metabolic health benefits of circuit training for older individuals with sarcopenia and obesity, thereby making a substantial contribution to the academic field.

As discussed above, sarcopenic obesity in older adults, a consequence of aging, is a significant factor leading to increased body fat percentage, reduced muscle mass and strength, hypertension, impaired vascular function, and heightened inflammation. However, there is a notable lack of long-term studies assessing the effects of exercise training on body composition, blood pressure, endothelial function, and inflammatory markers, which are key indicators of obesity in older people with sarcopenic obesity. Consequently, the aim was to verify the effectiveness of long-term circuit training in improving blood pressure, vascular function, and inflammatory markers in older obese women with sarcopenia.

## 2. Materials and Methods

### 2.1 Participants

This study targeted 30 older women, aged 65 or above, with sarcopenia and obesity living in S city. They were randomly assigned to either a control or exercise group, with 15 participants each. The sample size was calculated based on the FMD from a prior study by Yasuda *et al*. [27], yielding an effect size of 0.325 using Cohen’s method. With an alpha of 0.05, a power (1-β) of 0.8, 2 groups, and 2 repeated measures, the calculated sample size using G-power was 22. To accommodate for potential dropouts, 15 participants were selected for each group. Throughout the study, one individual from each group voluntarily dropped out due to personal reasons, resulting in 14 participants in each group.

Inclusion criteria were based on the diagnosis of sarcopenia (‘appendicular skeletal muscle mass/${\mathrm{height}}^{2}$’ less than 5.4 ${\mathrm{kg/m}}^{2}$) [28] and obesity (body fat percentage over 30%) [29], along with the ability to voluntarily participate without musculoskeletal disorders. Exclusion criteria included individuals who had participated in regular exercise programs more than twice a week within the last 6 months or had experienced serious clinical diseases such as metabolic or cardiovascular illnesses. The study was conducted in accordance with the Declaration of Helsinki and received approval from the Institutional Review Board (7001355-202012-HR-411). Participants were recruited after obtaining informed consent regarding the study’s purpose and procedures. The physical characteristics of the study subjects are shown in Table 1.

**Table 1.** **Participants’ characteristics (mean ± SD)**.

Variables	EG (n = 14)	CG (n = 14)	*p*
Age (years)	78.14 ± 3.72	78.21 ± 3.72	0.960
Height (cm)	152.05 ± 4.55	153.66 ± 4.42	0.350
Weight (kg)	52.21 ± 5.16	53.66 ± 6.17	0.508
BMI (${\mathrm{kg\cdot m}}^{–\,2}$)	22.58 ± 1.97	22.67 ± 1.81	0.901
Fat mass (kg)	18.11 ± 3.78	18.48 ± 2.90	0.773
Percentage fat mass (%)	34.43 ± 4.08	34.38 ± 3.08	0.974
Free fat mass (kg)	32.98 ± 2.41	34.17 ± 4.08	0.354
ASM (${\mathrm{kg\cdot m}}^{–\,2}$)	5.24 ± 0.29	5.18 ± 0.59	0.719

Note. SD, standard deviation; CG, control group; EG, exercise group; BMI, body mass index; ASM, appendicular skeletal mass.

### 2.2 Study Design

All participants were instructed to maintain a fast for at least 8 hours and to abstain from any medication before coming to the laboratory. Upon arrival, they were allowed to rest for 30 minutes before starting the tests. The examination began with blood pressure measurement, followed by body composition analysis, vascular elasticity and endothelial function assessments, and blood tests in that order. All tests were conducted by the same examiner using the same methods, both before and after the 12-week intervention period. The specific test items and methods were as follows.

### 2.3 Measurement

For assessing body composition variables, bioelectrical impedance analysis (BIA) using Inbody 770 (Korea) was employed to measure fat mass, fat-free mass, and percent body fat. Additionally, dual-energy X-ray absorptiometry (DEXA) using Primus, Osteosys (Korea) was utilized to measure total body muscle mass, from which the appendicular skeletal muscle mass index (ASMI) was calculated.

To measure blood pressure variables, resting heart rate (HRrest) was determined using the palpation method over one minute. Systolic blood pressure (SBP) and diastolic blood pressure (DBP) were measured using a mercury sphygmomanometer (SK, Welch Allyn Company, Jungingen, Germany). From the measured heart rate, systolic and diastolic blood pressures, mean arterial blood pressure (MAP = DBP + (SBP - DBP)/3), pulse pressure (PP = SBP - DBP), and rate-pressure product (RPP = HR × SBP) were calculated.

Brachial–ankle pulse wave velocity (ba-PWV) was measured using the VP-1000 plus (Omron Healthcare, Kyoto, Japan) to assess vascular function. After the subjects rested supine for 5 minutes, cuffs with attached sensors were wrapped around both arms and ankles to obtain waveform data. These data were used to measure the arterial stiffness of the brachial and posterior tibial arteries. For arterial endothelial function, a non-invasive Doppler ultrasound system (UNEX EF-38G, UNEX Co. Ltd, Nagoya, Japan) was used to measure FMD of the brachial artery. A cuff was placed on one arm for blood pressure measurement and another on the opposite forearm to occlude the brachial and forearm circulation. The ultrasound device was secured to an area 3–5 cm above the elbow of the brachial artery. Resting vascular diameter was measured using Doppler at the medial brachial artery. After measurements, the cuff was inflated to 50 mmHg above resting blood pressure to halt blood flow for 5 minutes. The diameter and blood flow speed were measured for 2 minutes after deflation. FMD was calculated using the formula: ((peak diameter – baseline diameter)/baseline diameter) × 100.

A trained nurse collected 8 mL of venous blood from the antecubital vein using a syringe to analyze inflammatory markers. The collected blood was placed into anticoagulated and untreated tubes according to the respective analysis requirements and centrifuged at 3000 rpm for 10 minutes to separate the plasma and serum from cellular elements. The separated plasma and serum were stored in storage tubes at –70 °C in a deep freezer until analysis. Subsequently, the frozen or refrigerated plasma, serum, and whole blood were sent to Seegene Medical Foundation’s clinical laboratory for analysis and disposal. The specific analysis methods for various blood components are as follows: High-sensitivity C-reactive protein (hs-CRP) was analyzed using the BNTM System (high-sensitivity CRP, Dade Behring, Marburg, Germany) through particle-enhanced immunonephelometry. TNF-α was analyzed using an ELISA kit (Biosource International, Camarillo, CA, USA). IL-6 was measured using a cytokine measurement ELISA kit (Biosource International, Camarillo, CA, USA).

### 2.4 Exercise Program

The circuit training program implemented in this study consisted of ten exercises: Walking in place, shoulder presses and squats, twist dashes, lunges, jumping jacks, kickbacks, modified push-ups, crunches, hip bridges, and bird dogs. Each session included 10 minutes of warm-up and cool-down exercises, with the main workout consisting of each exercise performed for 1 minute, totaling 10 minutes, followed by a 5-minute rest. The regimen was structured progressively over the 12 weeks: 2 sets for 25 minutes in weeks 1–2, 3 sets for 40 minutes in weeks 3–8, and 4 sets for 55 minutes in weeks 9–12, with sessions conducted three times a week (Table 2). During the exercises, participants wore a Polar H10 heart rate monitor (Polar Electro Oy, Kempele, Finland) to ensure that the exercise intensity corresponded to 60–85% of their heart rate reserve (HRR). Meanwhile, participants in the control group were instructed to maintain their usual lifestyle throughout the same period.

**Table 2.** **Circuit exercise training program**.

Stage	Mode	Time	Intensity
Warm-up	Stretching	10 minutes	HRR 60–85% 1 set (10 minute) Rest (5 minute) 3 times/week
Main	1–3 weeks: 2 sets	25 minutes	
	4∼8 weeks: 3 sets	40 minutes	
	9–12 weeks: 4 sets	55 minutes	
Cool-down	Stretching	10 minutes	

HRR, heart rate reserve.

### 2.5 Statistical Analysis

In this study, data analysis was conducted using SPSS (Statistical Package for the Social Sciences) version 25.0 (IBM Corporation, Armonk, NY, USA). The mean and standard deviation for all dependent variables were calculated. The Kolmogorov–Smirnov test was used to verify the normality of the distribution for all outcome variables. A repeated measure design two-way analysis of variance (ANOVA) was utilized to examine the interaction and main effects between the two groups before and after the exercise intervention. In cases where the group main effect, treatment main effect, or interaction between the group and treatment was significant, an independent *t*-test was conducted to analyze the mean differences in dependent variables between the two groups within the treatment. A paired *t*-test was used to verify the mean differences in dependent variables within the group between treatments. The significance level (α) for all statistical analyses was set at 0.05.

## 3. Results

Table 3 presents the results of changes in variables related to body composition, blood pressure, vascular function, and inflammation markers following a 12-week intervention. The content is as follows: Upon examining changes in body composition, interactions were noted in weight, body mass index (BMI), lean body mass, body fat mass, and body fat percentage. Notably, a significant increase in lean body mass was observed in the exercise group following 12 weeks of circuit training (*p* < 0.01). In the exercise group, both body fat mass and fat percentage significantly decreased (*p* < 0.001 and *p* < 0.01, respectively). Conversely, in the control group, significant increases were seen in body fat mass (*p* < 0.05) and body fat percentage (*p* < 0.05).

**Table 3.** **Changes in related variables over a 12-week intervention (mean ± SD)**.

Variables	EG			CG			*p* (*$\eta^{2}$*) value		
	Before	After	Mean change 95% CI	Before	After	Mean change 95% CI	Group	Time	Interaction
Body composition									
Weight (kg)	52.21 ± 5.16	51.61 ± 5.69	–0.60 [–1.32, 0.12]	53.66 ± 6.17	54.01 ± 6.12	0.35 [–0.02, 0.73]	0.387 (0.029)	0.521 (0.016)	0.018 (0.197)†
BMI (${\mathrm{kg\cdot m}}^{-2}$)	22.58 ± 1.97	22.28 ± 2.02	–0.30 [–0.61, 0.01]	22.67 ± 1.81	22.81 ± 1.79	0.14 [–0.03, 0.30]	0.670 (0.007)	0.330 (0.037)	0.013 (0.214)†
Fat mass (kg)	18.11 ± 3.78	17.00 ± 3.98	–1.11 [–1.51, –0.71]***	18.48 ± 2.90	19.48 ± 3.13	1.00 [0.27, 1.73]*	0.283 (0.044)	0.783 (0.003)	0.000 (0.536)†
Percentage fat mass (%)	34.43 ± 4.08	32.64 ± 4.24	–1.78 [–2.38, –1.18]***	34.38 ± 3.08	35.96 ± 3.19	1.58 [0.34, 2.82]*	0.238 (0.053)	0.756 (0.004)	0.000 (0.517)†
Free fat mass (kg)	32.98 ± 2.41	33.82 ± 2.52	0.84 [0.26, 1.43]**	34.17 ± 4.08	33.73 ± 4.01	–0.44 [–1.18, 0.30]	0.662 (0.007)	0.364 (0.032)	0.007 (0.249)†
ASM (${\mathrm{kg\cdot m}}^{-2}$)	5.24 ± 0.29	5.34 ± 0.26	0.10 [–0.02, 0.22]	5.18 ± 0.59	5.19 ± 0.54	0.01 [–0.06, 0.08]	0.517 (0.016)	0.079 (0.114)	0.173 (0.070)
Blood pressure related variables									
Heart rate (beats· $\min^{-1}$)	73.39 ± 7.43	67.89 ± 6.93	–5.50 [–10.60, –0.40]*	73.32 ± 11.62	74.46 ± 11.70	1.14 [–1.80, 4.09]	0.347 (0.034)	0.122 (0.089)	0.022 (0.186)†
SBP (mmHg)	133.79 ± 16.20	126.82 ± 8.90	–6.96 [–14.95, 1.02]	134.96 ± 13.03	134.00 ± 12.45	–0.96 [–3.89, 1.96]	0.358 (0.033)	0.054 (0.135)	0.139 (0.082)
DBP (mmHg)	80.39 ± 6.24	78.32 ± 7.89	–2.07 [–7.03, 2.89]	80.93 ± 11.47	81.25 ± 8.04	0.32 [–2.84, 3.48]	0.564 (0.013)	0.526 (0.016)	0.387 (0.029)
MAP (mmHg)	151.58 ± 19.94	142.99 ± 11.65	–8.60 [–18.47, 1.28]	152.98 ± 14.09	151.58 ± 14.34	–1.39 [–4.88, 2.10]	0.351 (0.034)	0.049 (0.140)†	0.149 (0.078)
PP (mmHg)	53.39 ± 11.69	48.50 ± 10.69	–4.89 [–11.72, 1.94]	54.04 ± 6.02	52.75 ± 6.80	–1.29 [–4.33, 1.76]	0.420 (0.025)	0.086 (0.109)	0.307 (0.040)
RPP	9856.14 ± 1841.47	8625.11 ± 1251.09	–1231.04 [–2003.98, –458.09]**	9922.80 ± 2044.46	9998.55 ± 1945.74	75.75 [–352.82, 504.32]	0.277 (0.045)	0.009 (0.235)†	0.004 (0.282)†
Vascular function related variables									
ba-PWV (cm/s)	1797.07 ± 201.12	1718.82 ± 215.67	–78.25 [–145.49, –11.01]*	1865.64 ± 159.23	1856.11 ± 159.77	–9.54 [–45.13, 26.06]	0.142 (0.081)	0.019 (0.193)†	0.062 (0.128)
FMD (%)	6.15 ± 1.34	7.39 ± 1.27	1.24 [0.79, 1.70]***	6.03 ± 1.12	5.83 ± 1.20	–0.20 [–0.54, 0.14]	0.071 (0.120)	0.001 (0.375)†	0.000 (0.535)†
Inflammatory cytokines									
*hs*-CRP (${\mathrm{mg\cdot L}}^{-1}$)	1.12 ± 0.88	0.80 ± 0.78	–0.32 [–0.87, 0.23]	1.10 ± 0.65	1.24 ± 0.77	0.14 [–0.03, 0.30]	0.426 (0.025)	0.491 (0.018)	0.098 (0.102)
IL-6 (${\mathrm{pg\cdot mL}}^{-1}$)	2.06 ± 1.04	1.55 ± 0.87	–0.51 [–0.80, –0.22]**	1.87 ± 1.04	1.97 ± 1.37	0.10 [–0.26, 0.45]	0.775 (0.003)	0.063 (0.126)	0.008 (0.242)†
TNF-α (${\mathrm{pg\cdot mL}}^{-1}$)	2.66 ± 1.48	2.46 ± 1.41	–0.21 [–1.01, 0.60]	2.50 ± 1.63	2.53 ± 1.53	0.03 [–0.61, 0.67]	0.925 (0.000)	0.718 (0.005)	0.625 (0.009)

Note. SD, standard deviation; CG, control group; EG, exercise group; BMI, body mass index; ASM, appendicular skeletal mass; SBP, systolic blood pressure; DBP, diastolic blood pressure; MAP, mean arterial pressure; PP, pulse pressure; RPP, rate pressure product; ba-PWV, brachial–ankle pulse wave velocity; FMD, flow-mediated vasodilation; hs-CRP, high sensitivity C-reactive protein; IL-6, interleukin 6; TNF-α, tumor necrosis factor-alpha; CI, confidence interval. † Significant interaction or main effect, *p < 0.05; **p < 0.01; ***p < 0.001 vs. before training.

Regarding blood pressure-related variables, significant interactions were noted in heart rate and RPP, with a main effect of time observed in MAP. Specifically, after 12 weeks of circuit training, the exercise group exhibited significant reductions in heart rate (*p* < 0.05) and RPP (*p* < 0.01).

Regarding vascular function-related variables, a main effect of time was evident in the ba-PWV, and both a main effect of time and an interaction were observed in the FMD. Specifically, after 12 weeks of circuit training, significant decreases in the ba-PWV (*p* < 0.05) and FMD (*p* < 0.001) were recorded in the exercise group.

As for changes in inflammatory markers, a significant interaction was observed in the levels of IL-6. Specifically, IL-6 levels significantly decreased in the exercise group after 12 weeks of circuit training (*p* < 0.05).

## 4. Discussion

The primary finding of this study is that circuit training, combining aerobic and resistance exercises, positively affects body composition, blood pressure, vascular function, and inflammatory markers in older obese women with sarcopenia. After 12 weeks of circuit training, there was a significant increase in fat-free mass and a notable decrease in fat mass and percent body fat. The influence of circuit training on body composition, particularly among older people, has been emphasized in recent research. A 12-week program combining resistance and aerobic exercise elements can significantly alter body composition by reducing body fat percentage and increasing lean muscle mass [25]. This is especially relevant for the aging population, where sarcopenia (loss of muscle mass and strength) and obesity are prevalent issues [30]. Circuit training, characterized by its variety and dynamism, promotes hypertrophy across various muscle groups and enhances overall physical function [31]. Additionally, the aerobic component of circuit training reduces visceral fat, lowering the risk of obesity-related diseases [32]. Prior studies, including Pieczyńska *et al*. [33], have reported that an 8-week program of combined exercises twice a week for 70 minutes effectively reduces body fat percentage. In contrast, Chen *et al*. [34] reported an increase in muscle mass but no significant difference in body fat percentage after a 12-week, twice-weekly, 60-minute exercise program in older obese with sarcopenia, partially aligning with the findings of this study. While an 8-week duration might be shorter but yield improvements, longer-term programs might be necessary. The 12-week duration with thrice-weekly sessions in this study is considered appropriate and potentially effective in improving the body composition of older obese women with sarcopenia.

Obesity is a multifactorial disease that can increase the risk of cardiovascular diseases. Exercise is recommended as a means of improvement [35]. In this study, heart rate and RPP significantly decreased after 12 weeks of circuit training, while MAP tended to decrease in the exercise group. Similar findings were reported in studies involving type 2 diabetes patients undergoing combined aerobic and resistance exercises for three months, showing significant reductions in HR and RPP [36]. Another study with older obese participants conducting combined exercises three times a week for 12 weeks reported significant decreases in SBP, MAP, and PP [26], which aligns with the results of this study. Exercise effectively lowers blood pressure by increasing arterial elasticity through the repetitive contraction and relaxation of arterial muscles. It is also associated with reduced myocardial load due to an increase in the number of capillaries surrounding muscle cells [37, 38]. Despite different subjects, when considering the positive changes in HR, RPP, and MAP with regular exercise in older people, a 12-week circuit training program also appears effective for older women with sarcopenic obesity.

In a meta-analysis examining the effects of exercise on blood pressure, it was reported that regular exercise alone did not produce significant changes in systolic or diastolic blood pressure among overweight and obese adults, while studies involving hypertensive patients suggested more noticeable effects from exercise [39]. In this study, the non-significant decrease in systolic blood pressure in the exercise group among older people with sarcopenic obesity and higher blood pressure levels suggests the need for alternative intervention approaches. Park *et al*. [40] reported significant decreases in systolic blood pressure following 24 weeks of combined aerobic and resistance exercises five days a week in older adults with sarcopenic obesity. Additionally, Barbat-Artigas *et al*. [41] found that a short-term combination of caloric restriction and aerobic exercise effectively reduced weight, body fat, and systolic blood pressure in older women with sarcopenic obesity. These findings suggest that longer-term exercise interventions or additional dietary approaches, such as caloric restriction, may be more effective in producing positive changes in blood pressure-related variables in older women with sarcopenic obesity.

The decrease in skeletal muscle due to aging is associated with independent risk factors for vascular diseases [42], and regular exercise is known to improve vascular endothelial function and increase vascular elasticity. This improvement is attributed to promoting nitrate (e.g., nitric oxide) production in vascular endothelial cells, contributing to vasodilation and improved blood flow [43]. The regular circuit training conducted in this study significantly improved ba-PWV and FMD. The vascular benefits of endurance training are generally related to relative intensity, suggesting that higher exercise intensity might lead to greater shear stress and noticeable vascular adaptations [44]. Park *et al*. [45] reported significant improvements in FMD among frail older women following an 8-week intervention of aerobic exercise, electrical stimulation, and protein-packed meals conducted three times a week. Indeed, prior studies indicate that a combination of aerobic and resistance exercises is associated with improvements or at least stabilization in arterial stiffness indicators in older adults and that combined exercise is identified as the most effective way to improve various cardiometabolic parameters in adults, especially those who are overweight or obese [46].

In vascular endothelium, nitric oxide (NO) is produced from L-arginine via endothelial nitric oxide synthase (eNOS), playing a crucial role in vasodilation [47]. According to Otsuki *et al*. [48], exercise can increase the bioavailability of NO and decrease ba-PWV. Similarly, El Assar *et al*. [49] reported that regular exercise can recover the reduced NO utilization due to oxidative stress and prevent endothelial dysfunction caused by aging. In this context, Kearney *et al*. [50] observed an increase in NO levels and a decrease in ba-PWV after 24 weeks of aerobic exercise, indicating a significant negative correlation between NO and ba-PWV. These findings collectively suggest that the circuit training conducted in this study could not only increase the synthesis and bioavailability of NO but also reduce circulating endothelin-1 (ET-1) levels, increase FMD of the brachial artery, improve endothelial function, and ultimately decrease ba-PWV. Therefore, it can be considered a positive exercise program for improving vascular function in older women with sarcopenic obesity.

This study showed no significant difference in CRP and TNF-α; however, a notable decrease was observed in IL-6 levels in the circuit training group. Previous studies have shown varied results: An 8-week kettlebell exercise program significantly reduced CRP levels in older adults with sarcopenia [51], and a 15-week combined exercise program significantly decreased TNF-α levels in older women with sarcopenia [52]. In contrast, Su *et al*. [53] reported significant improvements in both CRP and TNF-α after a 12-week combined exercise program in older individuals with type 2 diabetes, presenting a slight variance from this study. Rose *et al*. [54] stated that resistance exercise did not improve inflammatory markers, suggesting the importance of exercise intensity and volume to induce positive changes. IL-6 levels in patients with sarcopenia are independently associated with the development of sarcopenia and can promote catabolism in skeletal muscles, leading to muscle atrophy [55]. These findings indicate that combined exercise can enhance muscle strength and regulate IL-6 and TNF-α, thereby improving inflammatory markers. The decrease in TNF-α concentration might be attributed to increased muscle strength and mass due to resistance exercise [56]. Therefore, the circuit training conducted in this study might have been of relatively low intensity. It appears that higher intensity and longer exercise duration will be necessary to improve inflammatory markers significantly in the future.

In summary, the results of this study suggest that 12 weeks of circuit training, combining aerobic and resistance exercises, can be an effective method to improve body composition, blood pressure, vascular function, and inflammatory markers, thereby enhancing the health of older obese women with sarcopenia. However, it is important to note some limitations. The study might not have perfectly controlled for psychological and nutritional disruption factors throughout its duration and was limited to older women residing in the community. Future research should consider exploring varied intensities of resistance exercise and nutritional intake, among other interventions, for a more comprehensive understanding and application.

## 5. Conclusions

In this study, a 12-week circuit training program was investigated for its effects on blood pressure, vascular function, and inflammatory markers in older obese women with sarcopenia. The primary results indicate significant improvements in fat-free mass, fat mass, and body fat percentage in the exercise group. Blood pressure variables such as heart rate and RPP also showed significant improvements in the exercise group. Vascular function, measured by ba-PWV and FMD, improved significantly in the exercise group, and there was a notable decrease in IL-6 as an inflammatory marker. These findings suggest that circuit training can have a positive effect on improving body composition, blood pressure, vascular function, and inflammatory markers in older obese women with sarcopenia. These results imply that regular participation in a circuit training program can significantly enhance health-related variables in obese older adults with sarcopenia. To further confirm the wide-ranging effects of circuit training interventions on the health factors of older obese women with sarcopenia, more sophisticatedly designed further studies (e.g., varying intensity, duration, frequency, and combination of exercises) are necessary.

## Data Availability

The datasets used and/or analyzed during the current study are available from the corresponding author on reasonable request.
